# A Bayesian Modeling Approach to Optimize Longitudinal Biomarker Sampling Schedules Using Hormonal Data

**DOI:** 10.1002/ajhb.70302

**Published:** 2026-07-06

**Authors:** Monica H. Keith, Margaret Corley, Delaney J. Glass, Claudia Valeggia, Melanie A. Martin

**Affiliations:** ^1^ Department of Anthropology Vanderbilt University Nashville Tennessee USA; ^2^ Center for Studies in Demography and Ecology, University of Washington Seattle Washington USA; ^3^ Department of Ecology and Evolutionary Biology Yale University New Haven Connecticut USA; ^4^ Department of Anthropology Yale University New Haven Connecticut USA; ^5^ Department of Anthropology University of Toronto St. George Toronto Ontario USA; ^6^ Department of Anthropology University of Washington Seattle Washington USA

**Keywords:** Bayesian, biomarker, modeling, optimization, sampling

## Abstract

**Objectives:**

Optimal schedules for longitudinal biomarker sampling are specific to the individual biomarkers and study aims. We present a Bayesian modeling approach for evaluating intraindividual, interindividual, and population‐level biomarker variation in order to optimize precision in parameter estimates and inform biomarker sampling decisions.

**Methods:**

We apply Bayesian linear and nonlinear mixed‐effects models to estimate individual‐ and population‐level parameters of longitudinal hormone data from 35 pubertal girls. Starting with first morning void measures of urinary testosterone and C‐peptide collected across a two‐year timespan, we downsample these longitudinal data systematically to evaluate precision in parameter estimates from nine sampling frequencies: annual, biannual (6‐month), and quarterly (3‐month) intervals with one, two, and three repeated samples per interval.

**Results:**

Standard errors and credible intervals of individual‐ and population‐level parameter estimates as well as the overall residual errors from our applied models indicate that specific dimensions of sampling frequency have distinct impacts on model parameters across different levels. Collectively, metrics of model fit, precision, and uncertainty indicate that more data are not always better, as we do not find parameter estimates to improve directly with increasing total sample size across models. Notably, we identify optimal sampling thresholds beyond which individual parameter estimates become less precise with additional measures.

**Conclusions:**

The data, code, and results from these analyses provide tools for Bayesian model building, evaluation, and sampling decisions. Specific biomarker features impact precision in distinct ways, and our hormone modeling example showcases sampling analysis methods that are applicable to a broad range of biological data.

## 1 Introduction

Biomarker research balances concerns in measurement precision, accuracy, collection burden, and logistical limitations in sampling protocols, while researchers' abilities to devise and implement optimal sampling vary from study to study (Ferguson et al. 2014; O'Connor et al. 2006). Sampling designs that measure the same biological phenomenon may differ widely across studies with results gleaned from convenience samples, cross‐sectional data, or variable frequencies of repeated and longitudinal measures (Gildner 2021; Kenney et al. 2020). Sampling noise, error, and numerous dimensions of biological variability impact the ability to effectively capture variation both within and between individuals over time. Systematic analyses of these factors inform biologically meaningful interpretations from biomarker data and can be used to inform thresholds of biological significance (Faÿs et al. 2020; Nagaraj and Mann 2011; Subtil and Rabilloud 2014).

In this modeling methods paper, we utilize a series of intermittently sampled longitudinal datasets to evaluate measures of intraindividual, interindividual, and population‐level variation in pubertal hormone measures. Using approximately 2 years of testosterone and C‐peptide data from 35 pubertal girls, we model individual‐ and population‐level hormone trajectories in a Bayesian mixed‐effects framework by downsampling a longitudinal dataset with repeated measures at structured intervals.

This sampling analysis was originally conducted in order to inform subsample selection for a longitudinal cortisol analysis (Glass et al. 2024, 2025). Archived urine specimens had previously been assayed for testosterone and C‐peptide, and there was limited funding remaining to assay repeated measures of cortisol from the same samples. Therefore, our goal was to select an optimal subsample of measurements capturing hormonal variation within and between individuals to maximize longitudinal coverage using a minimum number of samples with low measurement error and uncertainty. We analyzed the existing longitudinal testosterone and C‐peptide measures to inform the subsequent cortisol sampling (Glass et al. 2024, 2025).

Here, we present our mixed model approach to determine sampling frequencies that optimize precision in capturing longitudinal biomarker parameters while also evaluating short‐term fluctuations in these urinary hormone data to inform the range of variability that may influence cross‐sectional measures. These analyses highlight descriptive and Bayesian inferential statistics for capturing intraindividual, interindividual, and population‐level variation in mixed‐longitudinal biomarker data.

### 1.1 Sources of Biomarker Variability

Biomarkers may respond to intrinsic and extrinsic exposures, vary in cyclical rhythms, trend directionally over longer periods of time, and fluctuate to varying degrees across different stages of the life course (Portaluppi et al. 2010). In this analysis, we use 2 years of longitudinal hormone data with repeated measures at quarterly (3‐month) intervals to assess intra‐ and interindividual variation in pubertal testosterone and C‐peptide. Hormone data such as these have a wide range of health applications, and puberty is an especially informative period for measuring growth, development, and energy allocation (Ellison 2017). Although the human endocrine system is highly conserved (Kleine and Rossmanith 2016), there is also high inter‐ and intraindividual variation in hormonal patterns (Williams 2007). Individual characteristics (e.g., stability of a hormonal relationship over time), biological rhythms, endocrine response to stimuli and exposures, sample measurement error (e.g., batch effects), and random sampling noise are among the myriad factors that may contribute to this variability. For example, measurement error may account for up to 5%–15% of the variation observed in hormonal samples and an average of 7.5% variability in urinary markers (Williams 2007; Nagaraj and Mann 2011).

The release of luteinizing hormone and follicle stimulating hormone during puberty prompts the production of steroids such as estrogen and testosterone, causing these hormone levels to rise over time (Ellison et al. 2012; Plant 2004). Concurrent with rising insulin resistance across puberty, basal insulin levels and C‐peptide (an insulin by‐product) are also expected to increase across puberty (Ellison 2017; Glass et al. 2024; Goran and Gower 2001; Hannon et al. 2006; Jeffery et al. 2012; Moran et al. 2002). Testosterone and other reproductive hormones also vary with ovarian cycling, and monthly fluctuations may add another dimension of intraindividual variability as pubertal individuals approach and begin ovarian/menstrual cycling (Atukorala et al. 2022). In terms of daily cycling, testosterone varies diurnally with its highest basal values in the morning and its nadir in the evening to late night (Matchock et al. 2007). C‐peptide is thought to peak between morning and midday in adults, but it is contested whether this rhythm is circadian or ultradian, and whether it applies to prepubertal and pubertal individuals (Arslanian et al. 1990; Bolli and Gerich 1984; Marin et al. 1988; Nicolau et al. 1984; Simon et al. 1987).

In addition to rhythms that hormonal trajectories may follow for an individual across daily and other cycles, there may be variability between individuals in how slopes or trajectories are associated with each other on any given sampling day, across time, or in certain seasons (Glass et al. 2025; Marceau et al. 2015; Matchock et al. 2007; Santi et al. 2020; Shirtcliff et al. 2015; Stanton et al. 2011; Van Anders et al. 2006; Zakreski et al. 2018). Genetic and environmental influences may contribute more or less variation to hormonal values across the day or at different sampling points (Grotzinger et al. 2018; Hoekstra et al. 2006). Testosterone may be sensitive to and vary with the immediate social environment, modulating behavior via bidirectional, organizational, and activational effects (Gildner 2021; Granger et al. 2003; Schulz, Molenda‐Figueira, et al. 2009; Schulz, Zehr, et al. 2009; Schulz and Sisk 2016; Varlinskaya et al. 2013), and C‐peptide may be especially sensitive to dietary and nutritional changes (Hoogwerf and Goetz 1983). Puberty itself may also impact hormonal variability at any sampling point, given that many sex steroids and gonadotropins increase in amplitude leading up to puberty when diurnal rhythms become evident (Ankarberg and Norjavaara 1999; Matchock et al. 2007; Mitamura et al. 2000). Furthermore, individual pacing of puberty and growth adds another source of intra‐ and interindividual variation in these measures across time and age. Human biologists must be attuned to these myriad sources of variability in order to effectively capture specific signals from such noisy biomarker data.

### 1.2 Sampling Considerations for Measuring Biomarker Variation

Optimal sampling designs are informed by intrinsic and extrinsic sources of variability and tailored to specific biomarkers and research aims. For example, cortisol is strongly influenced by day‐to‐day changes (including variations in sleep, stress, and physical activity) and minute‐to‐minute conditions; therefore, it is generally recommended to sample individuals across multiple days (Segerstrom et al. 2014). For ovarian hormones, sampling is scheduled around known fluctuations in ovarian cycling and would vary across aims to capture average hormone levels within a particular cycle phase, or to predict the timing of ovulation, and so forth (O'Connor et al. 2006).

Biomarker data used to predict a specific event (e.g., ovulation) can be evaluated for both *accuracy* (proximity of modeled predictions to actual events) and *precision* (proximity of measures to one another), whereas it is often possible to evaluate only precision or consistency in longitudinal data used to estimate trends over time (e.g., growth curves). Precision also varies according to specific study aims and applications. For example, measuring height daily may be appropriate for tracking short‐term growth rates during puberty (Hermanussen 2013, 2016, 2018); however, there is no precision benefit to sampling at intervals shorter than 1 year to capture long‐term growth curves (Cole 2018).

Prior research on testosterone and C‐peptide in puberty shows a wide range of sampling methods, frequencies, and research aims. As background research for this project, we conducted a random, nonsystematic review of existing literature (*n* = 36 articles) that sampled female testosterone and/or C‐peptide/insulin in adolescence to further contextualize our sampling case example in relation to how researchers have previously chosen to sample in related study designs (Table S1). We identified papers that assessed hormonal variation in puberty using Google Scholar and PubMed and summarized information relating to the original study scope, the hormonal specimen (C‐peptide/insulin or testosterone), whether the study was longitudinal or cross‐sectional, the reported sampling frequency and depth, as well as the sampling strategy and inclusion criteria (Table S1). Across these studies, 24 utilized longitudinal sampling designs, whereas 15 sampled cross‐sectionally; 8 included measures of C‐peptide, 22 included measures of testosterone, and 17 included measures of insulin. Among longitudinal studies that sampled testosterone, it was most common for researchers to sample once per occasion or to take multiple samples per measurement day (likely to capture diurnal response). Across studies that measured insulin or C‐peptide longitudinally, the most common strategy was to sample via fasting serum following standard insulin protocols, including multiple clamps or infusions, or to measure non‐fasting serum on more than one occasion across a study (Table S1). Authors across these studies highlight the need to systematically assess biomarker variation within and between individuals to inform and contextualize results, also speaking to challenges with variability and noise in pubertal hormone measures (e.g., Ankarberg and Norjavaara 1999; Biro et al. 2019; Cuartero et al. 2007; Kelly et al. 2011; Matchock et al. 2007).

Repeated sampling is needed over different time frames to discern between variation in hormone measures due to individual sources of variability (e.g., acute, diurnal, cyclical, and longitudinal changes within individuals over time), interindividual variation (e.g., differences in average, basal levels or in the magnitude of longitudinal changes between individuals), and unexplained variation due to potential measurement error and random noise. Repeated measures capture intraindividual variation, and longitudinal sampling increases intraindividual sampling depth which may improve measurement error. On the other hand, increasing repeated measures place additional burdens on participants, researchers, and resources, and they may also potentially increase sampling noise beyond optimal frequency thresholds for specific biomarkers. For studies aiming to capture meaningful biomarker differences between individuals, longitudinal sampling should be structured to sufficiently capture individual signals yet minimize endogenous variability in repeated measures.

As researchers balance both statistical considerations and logistical constraints, statistical power analyses often determine study sizes and sampling designs (Lai et al. 2003). However, relationships between statistical significance and biological significance may be disparate, as statistically significant results that detect an effect with sufficient confidence may not necessarily have an effect of sufficient magnitude to produce meaningful differences in health or biology (Nakagawa and Cuthill 2007; Kramer et al. 2016). Therefore, biological factors and metrics of variability must also be considered to critically inform biomarker sampling designs and interpretations (Smolders et al. 2014; Nagaraj and Mann 2011; Shvetsov et al. 2009).

### 1.3 Statistical Considerations for Measuring Biomarker Variation

Analyses of urinary proteomes have shown nearly comparable variation across intraindividual (45.5%) and interindividual (47.1%) dimensions of repeated data, highlighting the need to consider ranges of variation both within and between individuals over time (Nagaraj and Mann 2011). Statistical indicators of intraindividual variability include intraindividual standard deviations and coefficients of variation that capture the amplitude of fluctuations among an individual's measures across unordered timepoints, as well as autocorrelations and autocovariances that capture the temporal dependency of an individual's measurements and the extent to which a subsequent value varies according to their previous observations (Chrzanowski‐Smith et al. 2020; Wang et al. 2012). Composite measures such as the *mean square successive difference* incorporate elements of both temporal dependency and the amplitude of fluctuations but are limited in their ability to distinguish between these different dimensions of variability for individuals over time. Autoregressive coefficients can be produced by modeling individual linear trends or nonlinear curves through data points in a time series, from which individual trend parameters such as intercepts, slopes, and splines can be extracted for downstream application (Wang et al. 2012). Here, we apply this latter modeling approach to fit individual trend lines with varying slopes, intercepts, and splines to capture longitudinal biomarker trends and measure multiple aspects of intraindividual variability and precision in parameter estimates.

The magnitude of intraindividual variation can inform significance thresholds of intraindividual change or interindividual difference, and measures of interindividual variation are used to establish population‐level biomarker ranges and thresholds (Aziz et al. 2019; Chrzanowski‐Smith et al. 2020; Sobas et al. 2016). Summary statistics of variation across individuals include interindividual coefficients of variation, standard deviations, and composite measures such as the *index of individuality* that assess interindividual variation relative to intraindividual variance (Badrick 2021). Here, our mixed‐effect models estimate both individual‐ and population‐level pubertal trends in urinary testosterone and C‐peptide over time by modeling age as both a fixed (population‐level) and random (individual‐level) effect. Fit with Bayesian inference, the estimated errors and credible intervals from these models characterize probability distributions for all variables and directly measure precision and uncertainty around the parameter estimates derived from our series of sampling datasets.

Bayesian versus frequentist approaches are an additional statistical modeling consideration. Parameter coefficients estimated from Bayesian algorithms are derived from posterior distributions that yield direct measures of probability and uncertainty, in contrast to point estimates derived from frequentist models that are assumed to have one “true” fixed value with no underlying probability (A. M. Ellison 2004; Subtil and Rabilloud 2014; Van Zyl 2018). Foundationally, Bayesian models test the likelihoods of estimated parameter values across probability distributions given the observed data, whereas frequentist models inversely test the probability of the observed data having occurred given the hypothesized parameters. Bayesian credible intervals capture the probabilities in parameter estimates and ranges that frequentist confidence intervals are often assumed to reflect, and yet the latter is unable to quantify properties of parameter coefficients in this manner (Morey et al. 2016). Bayesian standard errors also directly capture variability in parameter estimation from the posteriors.

### 1.4 Study Aims

**FIGURE 1:**
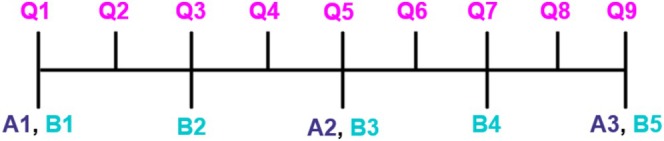
Structure of quarterly biomarker data downsampled to biannual and annual intervals. Nine sampling datasets capture 2 years of testosterone and C‐peptide measures across quarterly (3‐month), biannual (6‐month), and annual (1‐year) intervals with sampling depths of 1, 2, and 3 samples within the first month of each interval.

We present a modeling approach used to assess individual‐ and population‐level variation in urinary testosterone and C‐peptide data for informing sampling designs to optimize precision. Given our primary goal of assessing sampling frequencies in mixed‐longitudinal data to optimize precision and minimize uncertainty in parameter estimates, we fit mixed‐effects models with Bayesian inference and utilize statistics from the posterior distributions to compare model precision across different sampling frequencies. From an original mixed‐longitudinal dataset with 2 years of repeated testosterone and C‐peptide measures collected at quarterly (3‐month) intervals, we modeled individual‐ and population‐level hormone trajectories with datasets intermittently downsampled at quarterly, biannual, and annual intervals, and sampling depths of 1, 2, and 3 repeated measures per interval (Figure 1).

Analyzing these real‐world hormone data informs future sampling designs specific to these urinary biomarkers and also demonstrates longitudinal analysis methods that are applicable to a broad range of biological data. We evaluate multiple aspects of variation and precision based on model predictions, residuals, and estimated errors using Bayesian inference to obtain robust estimates of model parameters, their probabilities, and uncertainty (Van Zyl 2018). In this paper, we (1) assess monthly intraindividual variation in testosterone and C‐peptide to inform the range of potential variability to consider if using cross‐sectional or mixed‐longitudinal data, (2) assess intraindividual variability of predicted hormone estimates across different sampling frequencies to inform the magnitude of sampling noise that may obscure individual signals or interindividual differences, and (3) determine optimal sampling frequencies for these hormones that maximize precision and minimize error when modeling individual trajectories during puberty.

## 2 Materials and Methods

### 2.1 Data Collection and Hormone Assays

The data for these analyses were collected from the Chaco Area Reproductive Ecology (CARE) program in the periurban community of Namqom, in Formosa, Argentina, as part of a longitudinal study with perimenopausal women, pubertal girls, and mother‐infant dyads aimed at describing somatic, developmental, and endocrine biomarkers during key female life history transitions (NSF BCS‐0952264). A total of 61 girls aged 7–10 were recruited to participate in the study between 2011 and 2013, constituting all known premenarcheal Qom/Toba females residing in the area at the time. Every 3 months, participants were asked to provide first morning void urine samples twice a week over the course of 1 month (8 samples/month every 3 months, for a total of 32 samples maximum per year). This sampling schedule was optimized to capture ovarian cyclicity and variation in the perimenarcheal study participants following O'Connor et al. 2006, but duplicated for the girls to maximize coverage of hormonal maturation and standardize collection procedures across all study participants. Pubertal participants were followed until reporting three menstrual events. The mean number of monthly observations per subject was 24 ± 11 with sampling variance due to age at initial observation, age at menarche/age at study exit, and intermittent absences. The present study uses a balanced subset (see below) of all available testosterone and C‐peptide results collected and analyzed from 51 participants who provided a minimum of 1 year and up to 3 years of urine samples (mean ± SD samples/subject = 43 ± 16).

Participants were given sterile specimen collection cups the day prior to planned first morning void collection. Specimens were collected from participants' homes the following morning, transported to a research base in the Formosa capital, and aliquoted in triplicate. Aliquots were frozen at −20°C until transport to the United States, where they were stored at −80°C in the Yale Reproductive Ecology Laboratory until hormone assays were conducted. A total of 2190 urine samples from the 51 participants were analyzed for testosterone and C‐peptide in 2017. Testosterone assays were conducted by MM and MG using commercial EIA kits (Testosterone DetectX, Arbor Assay) at the Yale Reproductive Ecology Laboratory. C‐peptide assays were conducted at the Harvard Reproductive Ecology Lab using commercial radioimmunoassay kits (HCP‐20K, Millipore). Final concentrations for testosterone and C‐peptide were adjusted for specific gravity (Miller et al. 2004; Singh et al. 2015). Results from the full testosterone and C‐peptide data analysis have been published elsewhere (Martin et al. 2025).

### 2.2 Sampling Data Sets

The longitudinal data for this sampling analysis was measured repeatedly on a quarterly schedule, and we binned urinary testosterone and C‐peptide samples into quarterly (3‐month) intervals by individual, starting from each individual's first sample collection. Based on the collection schedule, each person could have up to eight samples per quarter (sampled within a 1‐month period). However, these real‐world longitudinal data were unbalanced, and one of our inclusion criteria for this sampling study was a minimum frequency of three samples per quarter. Additionally, we modeled between 1 and 2 years of hormone data per individual, subsetting to include only quarters one through nine (Figure 1).

In order to assess the impacts of both sampling interval and depth on precision, we sliced these hormone data at quarterly, biannual, and annual intervals. After downsampling to include only three samples per quarter for each person, we dropped intermittent quarters of data to derive sets with biannual (Quarters 1, 3, 5, 7, and 9) and annual (Quarters 1, 5, and 9) sampling intervals (Figure 1). Slice sampling to depths of two and one sample(s) per quarter, per person across these three intervals produced nine sampling data sets of one, two, and three samples by quarterly, biannual, and annual intervals. In order to capture longitudinal trends at annual intervals, each individual had to have at least two annual quarters of data represented to qualify for these sampling data sets.

Thirty‐five girls met these sampling inclusion criteria (mean age 10 years, range 8–13), and they each have between three and eight quarters of data (mean 6.26) to capture their testosterone and C‐peptide profiles across a one‐ to two‐year timespan for this study (Figure S1). Although the number of quarters with data varies between individuals, the quarters represented remain consistent within individuals across each sampling interval such that a girl with qualifying data for four out of the five biannual quarters has the same four quarters captured with sliced sampling frequencies of three, two, and one sample per interval.

### 2.3 Sampling Models

We modeled pubertal testosterone and C‐peptide trends across age for 35 girls in a Bayesian mixed‐effects framework using the brms package in R v. 4.6.0 (Bürkner 2017; R Core Team 2026). We estimated individual hormone slopes and intercepts by modeling age as a random effect by each ID in a series of linear mixed models (LMMs). This random effect structure estimated independent intercepts and slopes for each individual's linear hormone trends across their observed age range. Age was also included as a linear fixed effect in all LMMs, estimating the population‐level testosterone and C‐peptide profiles for pubescent Qom/Toba females across the sample's range from 8 to 13 years (1).

(1) $$\text{hormone}\;\left(\mathrm{ng}/\mathrm{ul}\right)\sim 1+\mathrm{age}+\left(1+\mathrm{age}|\mathrm{ID}\right)$$

LMMs implemented weakly regularizing priors on the population‐level age intercepts [normal(0,10)] and slopes [normal(0,1)] as well as the individual‐level standard deviations [cauchy(0,1)] (Lemoine 2019). Preliminary model fitting indicated that correlations between random‐level ages and IDs were negligible, thus we increased the correlation prior eta parameter to two [lkj(2)] in order to make more extreme correlations less likely and facilitate model fitting (Bürkner 2017).

Additionally, we ran a series of nonlinear models that fit both population‐ and individual‐level age trajectories using penalized regression splines with the package default weakly regularizing priors (Bürkner 2017). This flexible spline approach extends the LMM structure detailed above to generalized additive models (GAMs), allowing hormone trajectories to vary nonlinearly across time/age at both the individual‐ and population‐level (2). We chose a conservative, exploratory approach, in which the degree of smoothing is estimated from the data, allowing the effective flexibility of the spline to be determined empirically, rather than predetermined. In the model formula below s(age) captures the population‐level trajectory and s(age, ID, bs = “fs”) is a factor‐smooth that allows individual‐level deviations from that trajectory.
(2)

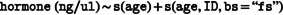




All models ran four chains for 20 000 iterations with a warm‐up of 10 000, and all effective sample sizes were > 5000 with Gelman‐Rubin statistics [Rhat] equal to 1.00, indicating successful chain convergence and robust sampling of the posteriors.

We ran nine LMMs and nine GAMs each for testosterone and C‐peptide to assess the impacts of varying sampling intervals and repeated measures within intervals on model parameter estimates. The individual‐ and population‐level pubertal hormone trends described above were estimated from quarterly, biannually, and annually sampled data with one, two, and three samples per interval (taken within a 1‐month period). This enabled us to assess intraindividual variation in modeled hormone trends and evaluate precision across different sampling frequencies. We evaluated precision in parameter estimates based on the magnitude of standard errors (standard deviations of posterior distributions for age effects that estimate linear and nonlinear hormone trajectories) and from the width of Bayesian credible intervals that contain probable ranges for each modeled parameter (Bürkner 2017; Van Zyl 2018). We were not able to evaluate accuracy based on “true” or “actual” values of parameter estimates, nor did we expect “precision” in repeated measures of testosterone and C‐peptide to cluster in a static manner within individuals in these mixed‐longitudinal data. Therefore, our goal was to identify sampling frequencies that minimize uncertainty or variability and maximize precision in parameter estimation of individual trends across age. The data and code for all analyses are included in Supporting Information Appendices A and B.

## 3 Results

**FIGURE 2:**
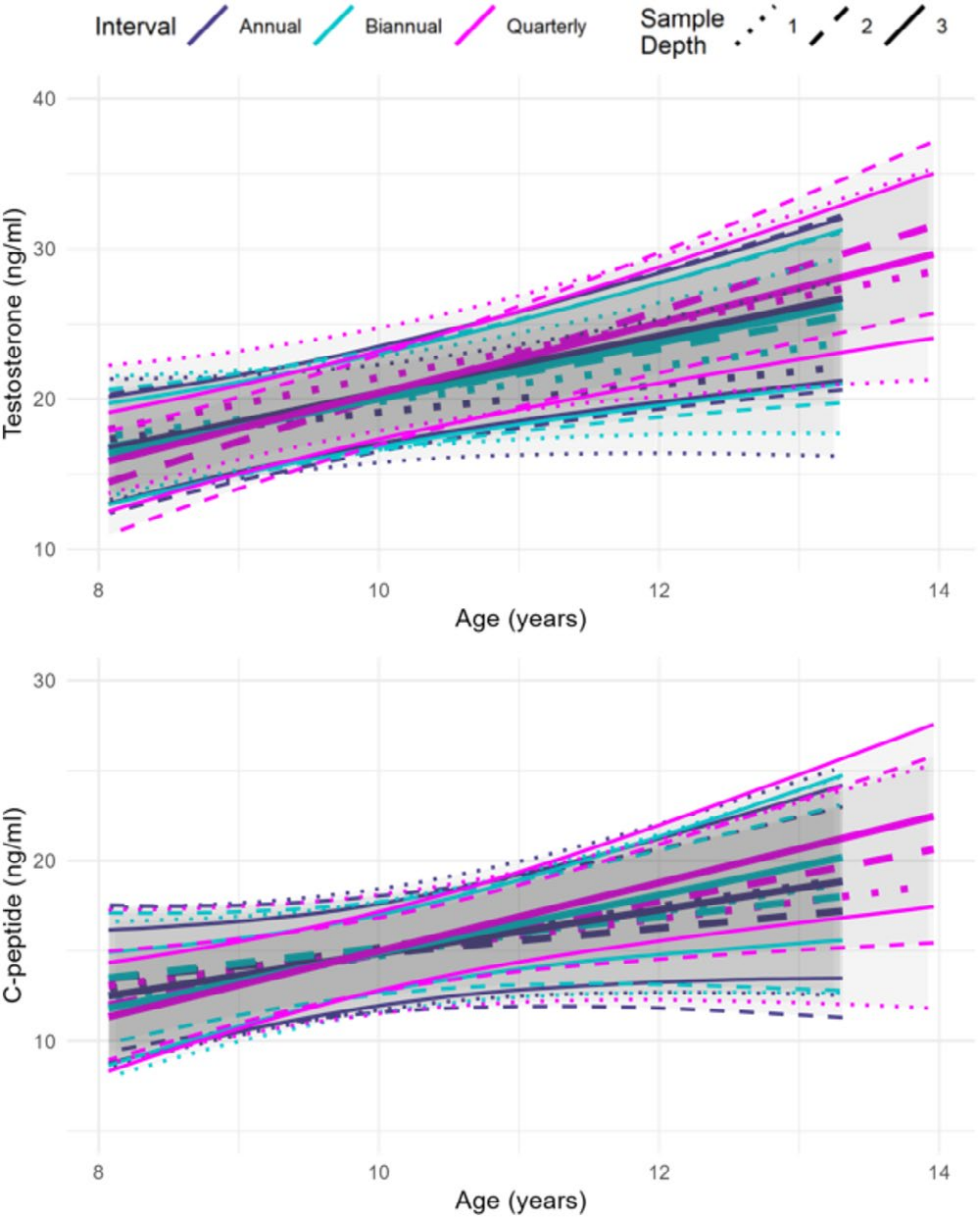
Population‐level estimates in testosterone (top) and C‐peptide (bottom) linear trends across age. Mean lines and shaded 95% credible intervals overlay estimates from nine linear mixed models with varying sample frequencies (quarterly, biannual, and annual intervals with 1, 2, and 3 samples per interval).

Monthly fluctuations in testosterone and C‐peptide levels increase in magnitude across age among pubertal Qom/Toba girls (Figure S2), as calculated from the difference between the minimum and maximum of three samples per participant within 1 month (Aim 1). Monthly intraindividual testosterone samples vary an average of 14 ng/mL, and the heteroskedasticity across age increases from an average range of 8 ng/mL at age eight to 20 ng/mL at 12 years (Figure S2). C‐peptide variability increases from 13 ng/mL at age eight to 23 ng/mL at age 12, averaging monthly fluctuations of 19 ng/mL across this pubertal age range overall. The magnitude of these short‐term fluctuations is greater than the linear increases observed in longitudinal trajectories across this pubertal age range (Figures 2 and S2; see Figure S1 for raw data points plotted per individual over time for further evidence of intraindividual variability). Figure S4 magnifies these features by showing raw observed data points from one individual with their LMM‐estimated trends overlaid. In Figure 2, depicting population‐level estimates of linear longitudinal hormone trajectories from our nine sampling LMMs, the average increases of approximately 12 ng/mL in testosterone and 9 ng/mL in C‐peptide across the sample age range are also smaller in magnitude than the average monthly intraindividual variation observed in these measures.

The shaded 95% credible intervals in Figure 2 overlap in large proportions across these nine LMMs, indicating that longitudinal population‐level estimates are relatively robust to varying sampling depths and intervals for these biomarkers. The estimated errors (standard deviations of posterior parameter estimates) for the fixed intercepts and slopes indicate that quarterly intervals with three repeated samples, quarterly intervals with two repeated samples, and biannual intervals with three repeated samples yield the most precise population‐level estimates with the smallest fixed effect error terms (Table 1).

**TABLE 1:** Bayesian linear mixed model results from nine sampling frequencies of testosterone and C‐peptide.

Sampling frequency	*n*_ID_	*n*_obs_	Individual intercepts mean (range)	Individual intercept errors mean (range)	Individual slopes mean (range)	Individual slope errors mean (range)	Population intercept mean (95% CI)	Population intercept error	Population slope mean (95% CI)	Population slope error	Residual error
*Testosterone models*											
Annual 1	35	79	0.02 (−0.57, 0.96)	2.31 (1.98, 3.99)	0.06 (−0.93, 1.93)	0.48 (0.39, 0.61)	9.99 (−3.74, 23.93)	7.05	0.91 (−0.48, 2.27)	0.70	7.15
Annual 2	35	158	0.00 (−0.37, 0.67)	2.34 (1.97, 4.36)	0.10 (−1.16, 2.60)	0.45 (0.37, 0.59)	0.86 (−11.82, 13.71)	6.52	1.92 (0.64, 3.19)	0.65	8.22
Annual 3	35	237	−0.02 (−0.28, 0.41)	2.65 (2.10, 5.50)	0.10 (−0.99, 2.49)	0.43 (0.34, 0.63)	1.25 (−9.96, 12.56)	5.75	1.91 (0.76, 3.05)	0.58	8.23
Biannual 1	35	129	0.02 (−0.75, 1.19)	2.33 (1.96, 4.30)	0.06 (−0.94, 1.87)	0.46 (0.38, 0.60)	8.06 (−6.31, 22.47)	7.30	1.17 (−0.25, 2.60)	0.72	9.02
Biannual 2	35	258	−0.01 (−0.20, 0.44)	2.26 (1.94, 4.00)	0.10 (−1.01, 2.54)	0.42 (0.34, 0.53)	3.45 (−8.82, 15.83)	6.29	1.66 (0.41, 2.89)	0.63	8.89
Biannual 3	35	387	−0.01 (−0.30, 0.64)	2.16 (1.83, 3.47)	0.08 (−1.07, 2.20)	0.36 (0.30, 0.48)	1.27 (−9.58, 11.97)	5.47	1.87 (0.78, 2.97)	0.55	8.79
Quarterly 1	35	219	0.04 (−0.99, 2.00)	2.90 (2.31, 5.29)	0.08 (−0.82, 1.76)	0.50 (0.41, 0.68)	3.65 (−11.19, 18.79)	7.64	1.78 (0.28, 3.24)	0.76	12.10
Quarterly 2	35	438	−0.02 (−0.29, 0.43)	2.07 (1.79, 3.14)	0.10 (−1.00, 1.91)	0.35 (0.30, 0.46)	−8.87 (−20.04, 2.50)	5.75	2.89 (1.75, 4.01)	0.58	9.35
Quarterly 3	35	657	0.02 (−0.79, 1.74)	2.51 (2.07, 4.89)	0.09 (−1.09, 2.11)	0.36 (0.29, 0.55)	−2.98 (−13.56, 7.55)	5.38	2.33 (1.28, 3.39)	0.54	10.32
*C‐peptide models*											
Annual 1	35	79	0.02 (−0.69, 1.93)	2.40 (2.09, 4.83)	0.04 (−0.51, 1.56)	0.50 (0.42, 0.86)	3.72 (−12.17, 19.92)	8.19	1.14 (−0.44, 2.70)	0.81	10.53
Annual 2	35	158	0.06 (−1.65, 5.83)	3.21 (2.62, 10.20)	0.03 (−0.63, 2.28)	0.48 (0.38, 1.11)	7.75 (−6.39, 22.06)	7.27	0.71 (−0.70, 2.10)	0.71	11.22
Annual 3	35	237	0.04 (−1.53, 4.50)	2.90 (2.28, 8.02)	0.03 (−0.60, 1.79)	0.43 (0.34, 0.89)	2.71 (−10.74, 16.18)	6.81	1.21 (−0.12, 2.55)	0.68	11.71
Biannual 1	35	129	0.01 (−0.49, 1.24)	2.19 (1.96, 3.44)	0.02 (−0.31, 0.87)	0.45 (0.38, 0.68)	2.52 (−13.45, 18.72)	8.13	1.22 (−0.36, 2.78)	0.80	13.59
Biannual 2	35	258	0.03 (−1.63, 3.95)	2.76 (2.25, 6.67)	0.02 (−0.53, 1.30)	0.39 (0.32, 0.76)	6.61 (−7.04, 20.38)	6.95	0.85 (−0.50, 2.20)	0.69	12.32
Biannual 3	35	387	0.02 (−1.64, 2.70)	2.45 (2.03, 4.42)	0.02 (−0.46, 0.77)	0.33 (0.28, 0.52)	−1.05 (−13.36, 11.29)	6.30	1.59 (0.37, 2.81)	0.62	12.68
Quarterly 1	35	219	0.05 (−1.09, 4.15)	2.91 (2.32, 9.14)	0.05 (−0.69, 2.64)	0.49 (0.40, 1.08)	5.48 (−9.55, 20.41)	7.65	0.94 (−0.53, 2.41)	0.75	12.39
Quarterly 2	35	438	0.02 (−1.90, 2.27)	2.44 (1.95, 3.77)	0.02 (−0.53, 0.69)	0.32 (0.26, 0.47)	0.18 (−11.80, 12.23)	6.11	1.46 (0.27, 2.66)	0.61	12.17
Quarterly 3	35	657	0.02 (−1.94, 4.15)	2.59 (1.99, 6.60)	0.03 (−0.64, 1.33)	0.33 (0.26, 0.71)	−3.92 (−15.31, 7.37)	5.78	1.89 (0.78, 3.02)	0.57	12.57

*Note:* Random effect statistics summarize 35 intercepts and slopes for each individual's hormone trend across age. Fixed effect statistics include 95% credible intervals (95% CI) for the estimated population‐level intercept and slope parameters of linear hormone age trends across all individuals. Intercept and slope errors reflect the standard deviation of each estimate's posterior distribution, and residual errors measure overall model fit and the magnitude of difference between observed data points and modeled predictions.

Next, we evaluated linear parameter estimates and hormonal predictions from our LMMs to address Aims 2 and 3. We predicted testosterone and C‐peptide levels for each girl at age 10 to inform the magnitude of sampling noise across our nine sampling frequencies (Aim 2). Testosterone estimates vary an average of 7 ng/mL, while C‐peptide estimates vary 6 ng/mL on average within individuals across our nine sampling LMMs (Figure S3). The magnitude of this sampling noise is less than both the monthly intraindividual variation described above and the population‐level longitudinal increases estimated from these data (Figures 2, S2, and S3).

**FIGURE 3:**
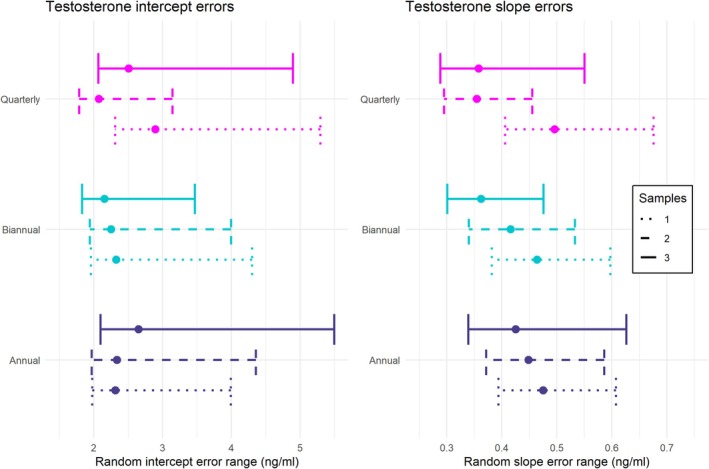
Means and full ranges of intercept (left) and slope (right) errors from individual‐level linear testosterone trends across age (*n* = 35) in nine models with varying sample frequencies (quarterly, biannual, and annual intervals with 1, 2, and 3 samples per interval).

**FIGURE 4:**
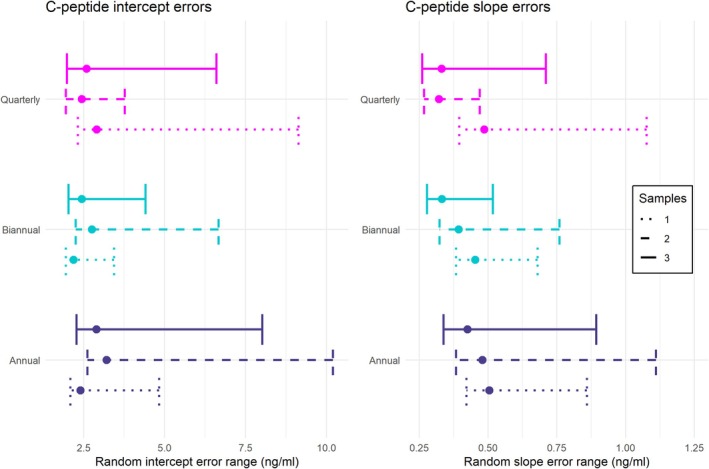
Means and full ranges of intercept (left) and slope (right) errors from individual‐level linear C‐peptide trends across age (*n* = 35) in nine models with varying sample frequencies (quarterly, biannual, and annual intervals with 1, 2, and 3 samples per interval).

We also evaluated precision in individual linear trend estimates by comparing random intercept and slope error ranges across these nine LMMs for the 35 Qom/Toba girls in this sample (Figures 3 and 4, Table 1). Random effect standard errors from these Bayesian LMMs measure uncertainty around estimated slopes and intercepts for each of 35 individual‐level hormone trends (Aim 3). We plotted the full distribution of 35 random effect standard errors across all nine sampling models for testosterone (Figure 3) and C‐peptide (Figure 4). For testosterone, quarterly sampling with two samples per interval and biannual sampling with three samples per interval show the smallest standard errors for individual intercepts and slopes (Figure 3). Both the averages and full ranges of 35 standard errors are lowest from these two sampling frequencies for random intercept and slope estimates (Table 1). Notably, quarterly sampling with three samples per interval is *less* precise, with higher averages and larger ranges of standard errors than several of the less frequent sampling LMMs. This differs from the pattern observed in our fixed effect results, as population‐level precision increases with more frequent sampling intervals in testosterone models with two and three repeated measures per interval but shows the opposite pattern across intervals sampled only once each (Table 1).

C‐peptide LMMs show the smallest standard errors in individual intercept estimates with two repeated samples at quarterly intervals, three samples at biannual intervals, and one sample at biannual intervals (Figure 4, Table 1). Quarterly 2‐sample and biannual 3‐sample LMMs show the lowest standard error averages and ranges for random‐level C‐peptide slopes, and these two sampling frequencies appear comparable and the most precise for capturing linear individual trends overall. Like testosterone, C‐peptide LMMs also show decreased precision (larger standard errors) in individual estimates with three repeated measures at quarterly intervals compared with several of the other less frequent sampling designs (Figure 4). Population‐level estimates of the linear C‐peptide trends show smaller standard errors with more repeated samples across a given interval, and across increasingly frequent intervals (Table 1).

In addition to the linear parameter results above, we evaluated precision and uncertainty in nonlinear trend estimates from a series of GAMs with the same nine sampling frequencies (Table S2). Figure S5 shows the estimated population‐level splines for testosterone and C‐peptide across this pubertal age range, and these flexible models produced approximately linear trajectories similar to the LMMs above. Largely overlapping 95% credible intervals indicate these nonlinear trends to be relatively robust to sample size, although population‐level testosterone splines show more variability across sampling frequencies than C‐peptide. Numeric output from these GAMs indicates that population‐level splines estimated from annual sampling with three samples per year have the lowest standard errors and best precision for both testosterone and C‐peptide, followed by annual sampling with two samples per year (Table S2). Individual‐level testosterone splines have the lowest standard errors in annual sampling with one sample per interval, whereas C‐peptide shows the best individual spline precision with quarterly sampling and two samples per interval (Figure S6). GAM residual error terms indicate that the best fitting splines overall are estimated from annual sampling with one sample per interval for testosterone and from quarterly sampling with two samples per interval for C‐peptide (Table S2).

Together, these model results characterize variability, sampling noise, and precision in Bayesian estimates of individual‐ and population‐level hormone trends. The mixed‐longitudinal data for these analyses were collected intermittently with repeated samples and show relatively high monthly variability in individual hormone levels, which influences precision when modeling linear and nonlinear longitudinal trajectories over longer timescales. Our analyses demonstrate a Bayesian approach for evaluating sampling frequency to optimize study designs while also assessing specific aspects of intra‐ and interindividual variation to inform biologically meaningful interpretations from the data.

## 4 Discussion

Our findings underscore the importance of tailoring sampling strategies to the biological properties of specific biomarkers, the medium in which they are collected, and intended downstream applications. For urinary testosterone and C‐peptide, which exhibit high short‐term intraindividual variability, our results suggest that intermediate sampling frequencies (two samples per quarter or three samples biannually) optimize precision for capturing linear longitudinal trends. Unlike ovarian hormones, for which optimal intermittent sampling protocols have been established (O'Connor et al. 2006), there is no consensus on longitudinal sampling frequency for testosterone or C‐peptide. Our sampling case example empirically evaluates how different sampling frequencies affect precision in modeling these hormones during a dynamic period of pubertal change.

We analyzed mixed‐longitudinal hormone data that were structured with repeated measures at quarterly (3‐month) intervals for properties of intraindividual, interindividual, and population‐level variation. Each participant had three repeated measures of testosterone and C‐peptide per month of data across a 1‐ to 2‐year timespan and was followed in the study through their first three menstrual events. The structure of these data enabled us to assess multiple aspects of sampling frequency (varying intervals and number of repeated measures within intervals) and the impacts of overall sample size on precision in model‐estimated parameters. Maximizing precision is a key aim of sampling optimization (Faÿs et al. 2020; O'Connor et al. 2006), and our mixed‐effects modeling approach demonstrates that systematically analyzing intra‐ and interindividual variation can both optimize sampling precision and inform thresholds of biological significance or difference (Nagaraj and Mann 2011).

### 4.1 Short‐Term Variability Impacts Measures Within and Between Individuals

Our first aim was to assess short‐term intraindividual variation with descriptive statistics of repeated measures, and these pubertal urinary hormone data show relatively high variability with monthly ranges averaging 14 ng/mL for testosterone and 19 ng/mL for C‐peptide across 35 participants aged 8–13 years. Figure S2 also shows increasing intraindividual variation in these repeated measures across age, indicating that monthly fluctuations in testosterone and C‐peptide increase in magnitude as girls approach menarche and begin menstrual cycling. Given that the monthly intervals for these repeated first morning void urine samples correspond in length generally to the monthly endocrine cycling of ovulation/menstruation (Bull et al. 2019), we infer that these monthly intraindividual ranges indicate biologically meaningful ranges within which an individual's cross‐sectional data may fall when only a single measurement is available. If using cross‐sectional data, analyses of interindividual differences should thus consider these intraindividual ranges as thresholds for biological significance or difference, as cross‐sectional differences that lie within the scope of intraindividual variation have no additional context to distinguish representative signals from cyclical variation, noise, and error (Badrick 2021; Burt et al. 2017; Chrzanowski‐Smith et al. 2020; Louis et al. 1986; Maxwell and Cole 2007).

Our second aim was to assess intraindividual variation in predicted hormone values across nine sampling designs of varied intervals and depths, and the resulting intraindividual ranges of LMM‐predicted hormone values at age 10 produced estimates of sampling noise (Figure S3). On average, testosterone estimates varied 7 ng/mL and C‐peptide estimates varied 6 ng/mL within individuals across our nine modeled sampling frequencies. These estimates can inform signal‐to‐noise ratios that may obscure individual hormone signals and should be considered alongside the monthly intraindividual variability discussed above when evaluating interindividual differences (Badrick 2021; Del Giudice and Gangestad 2022).

### 4.2 Intraindividual Variation Impacts Precision in Longitudinal Estimates

Our third aim was to determine sampling frequencies that optimize precision in capturing individual urinary testosterone and C‐peptide trends across age, and we focused primarily on evaluating linear parameter estimates for further downstream applications (Glass et al. 2024, 2025). The nonlinearly modeled hormone trajectories also appeared largely linear (Figure S5), consistent with prior analyses of these samples demonstrating that the pubertal inflection and leveling‐off of C‐peptide is not evident until later ages (Martin et al. 2025). These findings further support the use of linear modeling in future work using these hormones and at early to mid‐pubertal stages.

Standard errors from Bayesian models capture precision and uncertainty in estimated intercepts and slopes directly from the posterior distributions, and the estimates from sampling models with the smallest standard errors indicate the highest precision (Subtil and Rabilloud 2014). Notably, individual intercept and slope estimates show neither the most precision with the largest sampling datasets nor an overall association between total sample size and standard errors in either linear or nonlinear models (Figures 3, 4, and S6; Tables 1 and S2). Across the LMMs, testosterone sampled at quarterly intervals with two samples per interval shows the highest precision and smallest standard errors across these 35 individuals (Figure 3; Table 1). C‐peptide sampled at quarterly intervals with two samples, or at biannual intervals with three samples per interval, shows the smallest random‐level intercept and slope errors (Figure 4; Table 1). These sampling frequencies show lower errors and better precision than models sampled more frequently at quarterly intervals with three samples per interval, indicating that there is an optimal sampling threshold beyond which additional measures do not improve individual‐level model estimates or precision. Estimates of individual‐level splines from the GAMs provide additional support for this optimizing property, as most of the less frequent sampling designs yield better precision and less uncertainty in nonlinear parameters than the most frequent design with the largest sample size (Figure S6; Table S2). Furthermore, the total number of observations per individual across these sampling frequencies does not correspond directly to the magnitude of standard errors in individual spline estimates from the GAMs or in LMM random‐effect intercepts or slopes (Tables 1 and S2).

Although testosterone and C‐peptide levels are known to rise in individuals during puberty, these hormone markers are also highly variable on short‐term timescales, which introduces environmental and random noise in data from which we aim to extract longitudinal signals and trends (Shirtcliff et al. 2009; Ellison et al. 2012; Ellison 2017). Researchers across a multitude of disciplines (e.g., neuroimaging, bioinformatics, machine learning, engineering, epidemiology) have shown that the magnitude of noise in their respective datasets impacts their ability to detect meaningful signals, trends, and parameters in variable‐specific ways, as models increasingly struggle to parse signal from noise with more random or extraneous variation in the data (Ades‐Aron et al. 2018; Bellera et al. 2008; Nettleton et al. 2010; Vadillo and Garaizar 2016). Evaluations of statistical precision in parameter estimation and model prediction in these examples generally indicate that precision declines, uncertainty increases, and models increasingly struggle with prediction as noise increases, and many consequently advise pruning and processing data to decrease noise prior to model specification. This statistical context yields support for our sampling model findings in which testosterone and C‐peptide parameter estimates become less precise with additional data beyond “optimal” sampling frequencies.

Additionally, residual errors from our Bayesian models are indicators of fit reflecting how well predicted values that are based on model estimated parameters correspond with the observed data, and overall model fit does not appear to associate directly with sample size in either urinary testosterone or C‐peptide (Tables 1 and S2). Models sampled annually with only one measure per year have the lowest residual errors, and this fit statistic indicates that sampling interval and depth each have distinct impacts on overall model prediction. Including age as a fixed‐level effect in LMMs produced estimates of population‐level intercept and slope parameters for testosterone and C‐peptide trends over time, and standard errors for these population‐level terms provide another metric of model precision. While population‐level intercepts and slopes show the lowest standard errors with the largest sample sizes for both hormones (three samples per quarterly interval), this indicator does not associate directly with total sample size (Table 1). Rather, precision in population‐level parameters is impacted and improved more by increasing sampling depth (number of monthly repeated measures per interval) than by sampling interval or by total sample size in this case example.

Together, standard errors of individual‐ and population‐level parameter estimates as well as the overall residual errors from this series of Bayesian sampling models indicate that specific dimensions of sampling frequency and size have distinct impacts on model parameters across different levels. The least frequently sampled models with the lowest total sample size show the “best” overall fit in terms of residual error and model prediction, whereas estimates of population‐level parameters are more precise in the most frequently sampled models (Tables 1 and S2). Intermediate sampling frequencies show the best precision for estimating linear and nonlinear individual‐level parameters from these hormone data, and there appears to be an optimal threshold beyond which precision *decreases* with additional samples. Notably, no metric of model fit or precision shows a direct association with total sample size (Tables 1 and S2); therefore, we conclude that model estimates are not always improved with more data. Specific to particular study aims and biomarkers, there are optimal thresholds beyond which additional data may increase noise and obscure estimated signals, and these properties are influenced by the magnitude and dimensions of variation in the data.

### 4.3 Applications and Limitations

In practice, optimal sampling designs must balance data precision with logistical limitations and collection burdens. In longitudinal studies, more frequent data collection intervals may be less feasible to sustain over longer periods of time. The results from our sampling models here suggest that some trade‐offs between data precision and collection burden may be mitigated by collecting more repeated samples at longer, fewer intervals. For example, we found nearly identical precision in LMM estimates of individual hormone trends using three samples at biannual intervals (*n*_observations_ = 387) as with two samples at quarterly intervals (*n*_observations_ = 438).

In our sampling case example, the magnitude of monthly variability in these pubertal hormone data underscores the importance of collecting samples structured according to specific biomarker attributes. We found the averages of monthly intraindividual fluctuations (testosterone: 14 ng/mL and C‐peptide: 19 ng/mL) to exceed the average linear increases of 12 ng/mL for testosterone and 9 ng/mL for C‐peptide across the 8‐ to 13‐year‐old age range of these data (Figures S2 and S3). The outsized magnitude of short‐term variability captured in these repeated measures illuminates the noisiness of these particular urinary biomarkers and provides context for our longitudinal results which show that there is a threshold beyond which more samples *decrease* precision of individual parameter estimates. Moreover, our approach to measuring short‐term variability in repeated measures and sampling error across different sampling model predictions demonstrates how evaluating structured dimensions of variation can be applied to inform thresholds of biological change, significance, or difference.

The structured nature of the data in our case example allowed us to evaluate the precision effects of different sampling intervals and repeated measures within intervals, however, we were limited by the schedule on which these data were originally collected. We were not able to evaluate sampling intervals shorter than 3‐months or more than three repeated measures per interval, and there are intraindividual dimensions of variability in testosterone and C‐peptide that are not captured in these first morning void urine samples (e.g., diurnal variation). These sampling properties limit our scope for evaluating intraindividual, interindividual, and population‐level variation, however, our descriptive statistics and Bayesian inferences demonstrate applied methods for extracting individual signals from noisy biomarker data and contextualizing biologically meaningful differences and trends.

Our sampling model results highlight the utility of Bayesian inference for estimating parameter probabilities and uncertainties, and the corresponding R code shows how to extract model estimated predictions, residuals, fit statistics, and properties of posterior distributions from brms models (Supporting Information Appendix B). In contrast, frequentist models provide only a maximum likelihood point estimate for each parameter, which is the average value most likely to have produced the observed data, while the associated standard errors, confidence intervals, and other frequentist statistics are derived from the sampling distribution of observed data and are scaled for sample size (Morey et al. 2016; Van Zyl 2018). Frequentist estimates reflect long‐run expectations of repeated data collection with applications oriented around null hypothesis significance testing of modeled parameters, whereas Bayesian parameter estimates reflect probabilistic uncertainties across a range of possible values (Ellison 2004). Bayesian Markov Chain Monte Carlo (MCMC) algorithms sample full posterior probability distributions that integrate prior parameter probabilities with observed data, and the coefficient estimates, standard errors, credible intervals, and other Bayesian model statistics reflect sampled properties of likely parameter values. While formal theoretical and mathematical comparisons between frequentist and Bayesian inferences are beyond the scope of this sampling methods paper, we aimed to highlight applications of Bayesian inference for parameter estimation and evaluation by providing example interpretations, data, and code for our sampling results.

The data and code for our analyses can be used to reproduce our results and familiarize researchers with our statistical methods (Supporting Information Appendices A and B). Our code for data processing, downsampling, model syntax, parameter specification, and model evaluation could also be adapted to other datasets and exploratory aims oriented around parameter estimation. Collectively, our metrics of model fit, precision, and uncertainty indicate that more data are not always better, as we did not find parameter estimates to improve directly with increasing total sample size across models. Instead, specific biomarker features impact precision in distinct ways, and the sampling methods presented here can be applied to the evaluation and optimization of other biomarkers and sampling designs.

## Author Contributions

**Monica H. Keith:** conceptualization, formal analysis, methodology, software, visualization, writing – original draft. **Margaret Corley:** investigation, resources, writing – review and editing. **Delaney J. Glass:** investigation, writing – original draft. **Claudia Valeggia:** funding acquisition, investigation, project administration, resources, writing – review and editing. **Melanie A. Martin:** conceptualization, funding acquisition, investigation, project administration, writing – original draft.

## Funding

Support for this research came from the Center for Studies in Demography and Ecology (CSDE) at the University of Washington (P2C HD042828 and NIH T32 HD101442‐01) and the National Science Foundation (NSF BCS‐0952264).

## Ethics Statement

All girls and their adult caretakers provided verbal informed consent to participate. Consent was given at every sample collection. The research protocol was approved by the internal review boards of the University of Pennsylvania (Protocol #811200) and Yale University (HSC Protocol #1406014104 for original data collection and #2000026021 for ongoing data analysis).

## Conflicts of Interest

The authors declare no conflicts of interest.

## Supporting information


Appendix A.

Appendix B.

**Figure S1:** Individual line graph of mixed repeated hormone measures (top: C‐peptide, bottom: testosterone) over time by participant (*n*_observations_ = 657, *n*_individuals_ = 35).
**Figure S2:** Monthly intraindividual ranges in testosterone and C‐peptide by age. *n* = 35 girls with 219 repeated monthly ranges per hormone plotted with linear trend lines across all ranges.
**Figure S3:** Violins show ranges of predicted C‐peptide (left) and testosterone (right) for each girl at age 10 (*n* = 35) based on nine linear sampling models' fitted parameters.
**Figure S4:** Observed biomarkers (3 samples per quarterly interval, *n* = 27 testosterone and C‐peptide) for one individual across ages 8.5–10.5 years with linear mixed model‐estimated trends overlaid.
**Figure S5:** Population‐level estimates in testosterone (top) and C‐peptide (bottom) nonlinear trends across age.
**Figure S6:** Means and full ranges of testosterone (left) and C‐peptide (right) standard errors from individual spline estimates across age (*n* = 35) in nine nonlinear generalized additive models with varying sampling frequencies (quarterly, biannual, and annual intervals with 1, 2, and 3 samples per interval).
**Table S1:** Literature summary table of pubertal testosterone and C‐peptide/insulin studies. Overview of study scope, biomarkers collected, and sampling criteria.
**Table S2:** Bayesian generalized additive model results from nine sampling frequencies of testosterone and C‐peptide.

## Data Availability

All data and code for the presented analyses are included as Supporting Information Appendices A and B.
