# Supplementary material for: A Bayesian Modeling Approach to Optimize Longitudinal Biomarker Sampling Schedules Using Hormonal Data
Source: Am J Hum Biol. 2026 Jul 6;38(7):e70302. doi: 10.1002/ajhb.70302 (PMC13334439; doi:10.1002/ajhb.70302)
Supplement: Supplementary file 3 — Figure S1: Individual line graph of mixed repeated hormone measures (top: C‐peptide, bottom: testosterone) over time by participant (n observations = 657, n individuals = 35). Figure S2: Monthly intraindividual ranges in testosterone and C‐peptide by age. n = 35 girls with 219 repeated monthly ranges per hormone plotted with linear trend lines across all ranges. Figure S3: Violins show ranges of predicted C‐peptide (left) and testosterone (right) for each girl at age 10 (n = 35) based on nine linear sampling models' fitted parameters. Figure S4: Observed biomarkers (3 samples per quarterly interval, n = 27 testosterone and C‐peptide) for one individual across ages 8.5–10.5 years with linear mixed model‐estimated trends overlaid. Figure S5: Population‐level estimates in testosterone (top) and C‐peptide (bottom) nonlinear trends across age. Figure S6: Means and full ranges of testosterone (left) and C‐peptide (right) standard errors from individual spline estimates across age (n = 35) in nine nonlinear generalized additive models with varying sampling frequencies (quarterly, biannual, and annual intervals with 1, 2, and 3 samples per interval). Table S1: Literature summary table of pubertal testosterone and C‐peptide/insulin studies. Overview of study scope, biomarkers collected, and sampling criteria. Table S2: Bayesian generalized additive model results from nine sampling frequencies of testosterone and C‐peptide. [file AJHB-38-e70302-s001.docx]

SUPPLEMENTAL MATERIALS

**
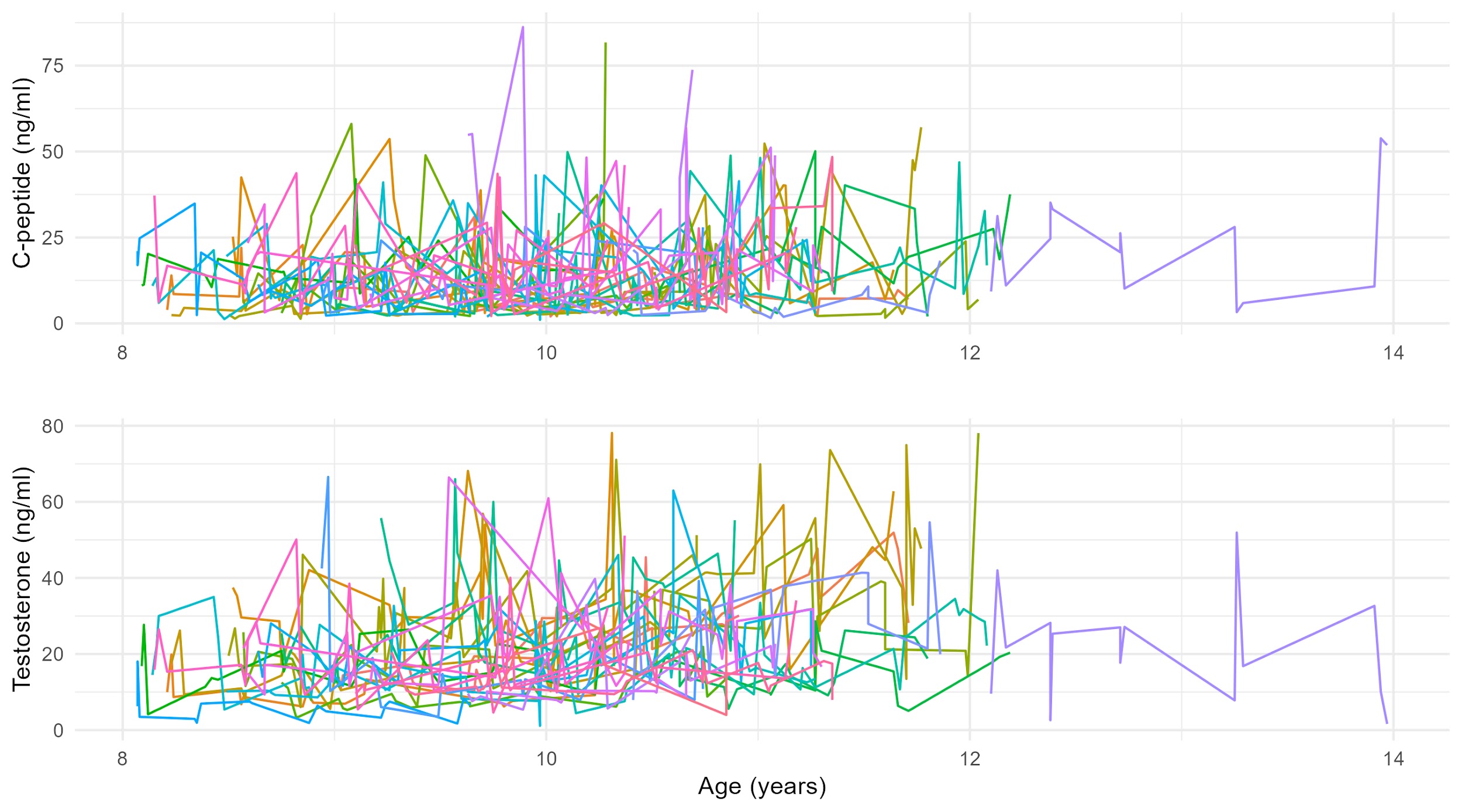
**

**Figure S1.** Individual line graph of mixed repeated hormone measures (top: C-peptide, bottom: testosterone) over time by participant (n_observations_=657, n_individuals_=35).

**
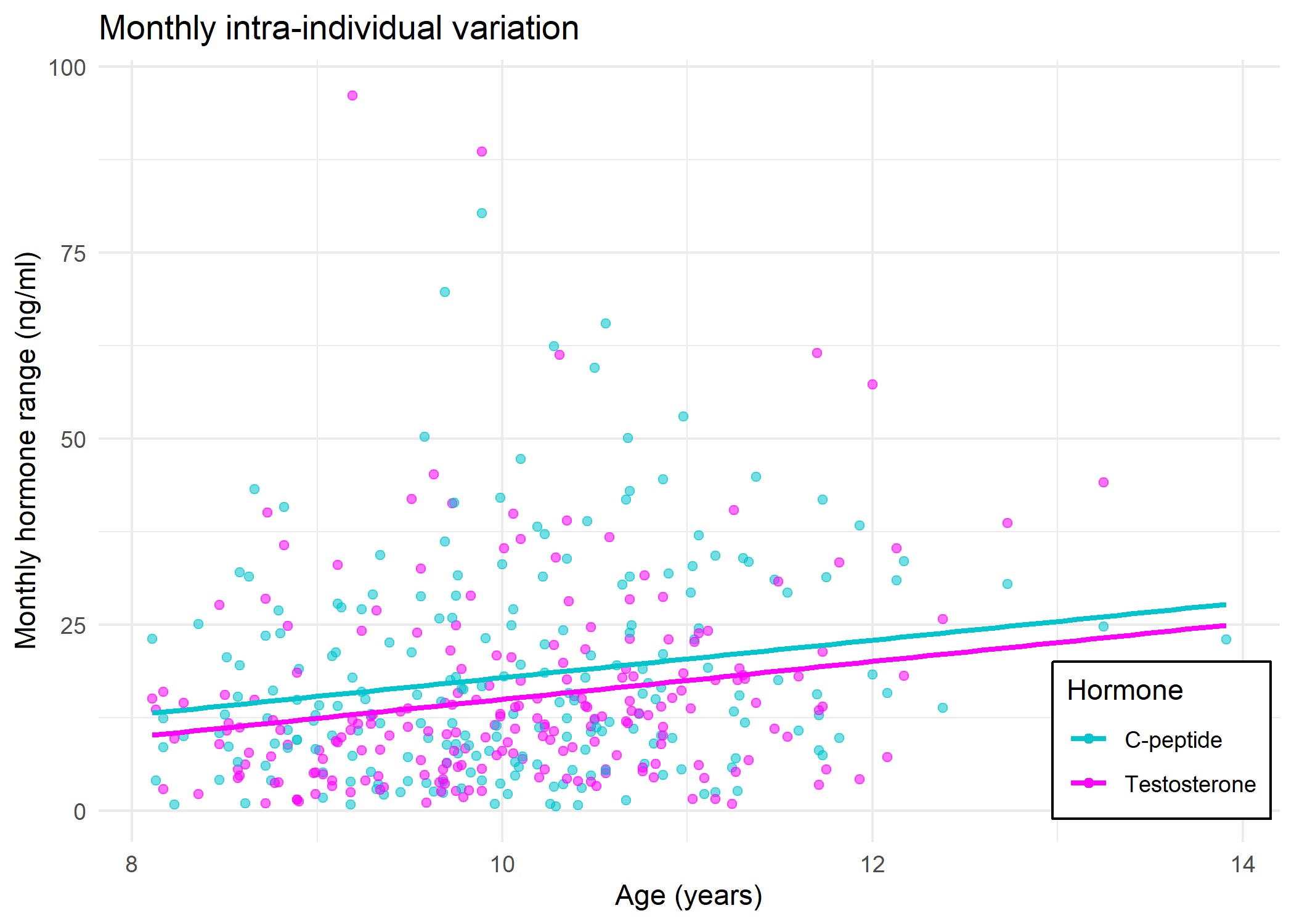
**

**Figure S2.** Monthly intra-individual ranges in testosterone and C-peptide by age. n=35 girls with 219 repeated monthly ranges per hormone plotted with linear trend lines across all ranges.

**
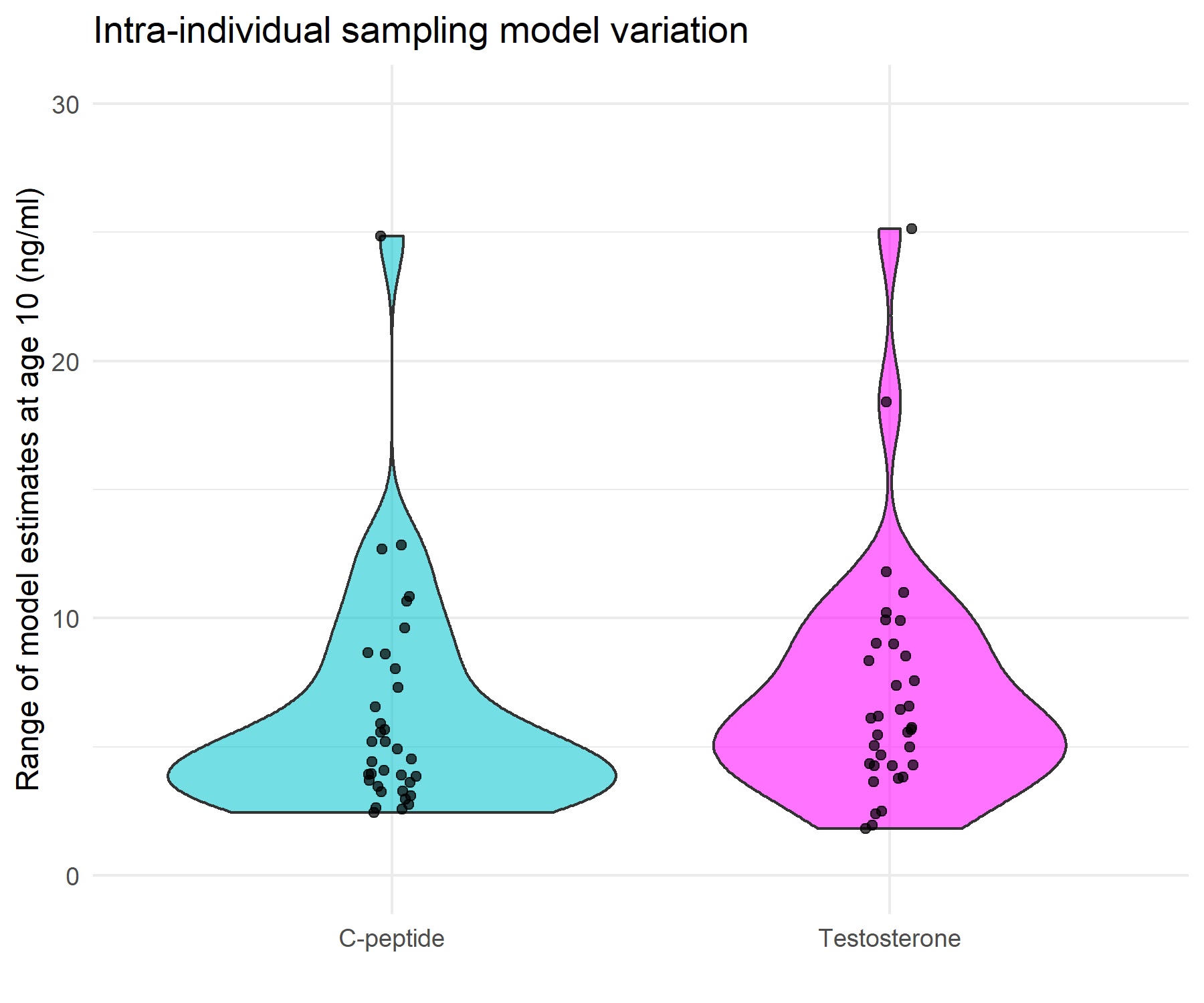
**

**Figure S3.** Violins show ranges of predicted C-peptide (left) and testosterone (right) for each girl at age 10 (n=35) based on nine linear sampling models’ fitted parameters. Each range reflects the difference between a girl’s minimum and maximum estimates across linear mixed models with samples at quarterly, biannual, and annual intervals and 1, 2, and 3 samples per interval.


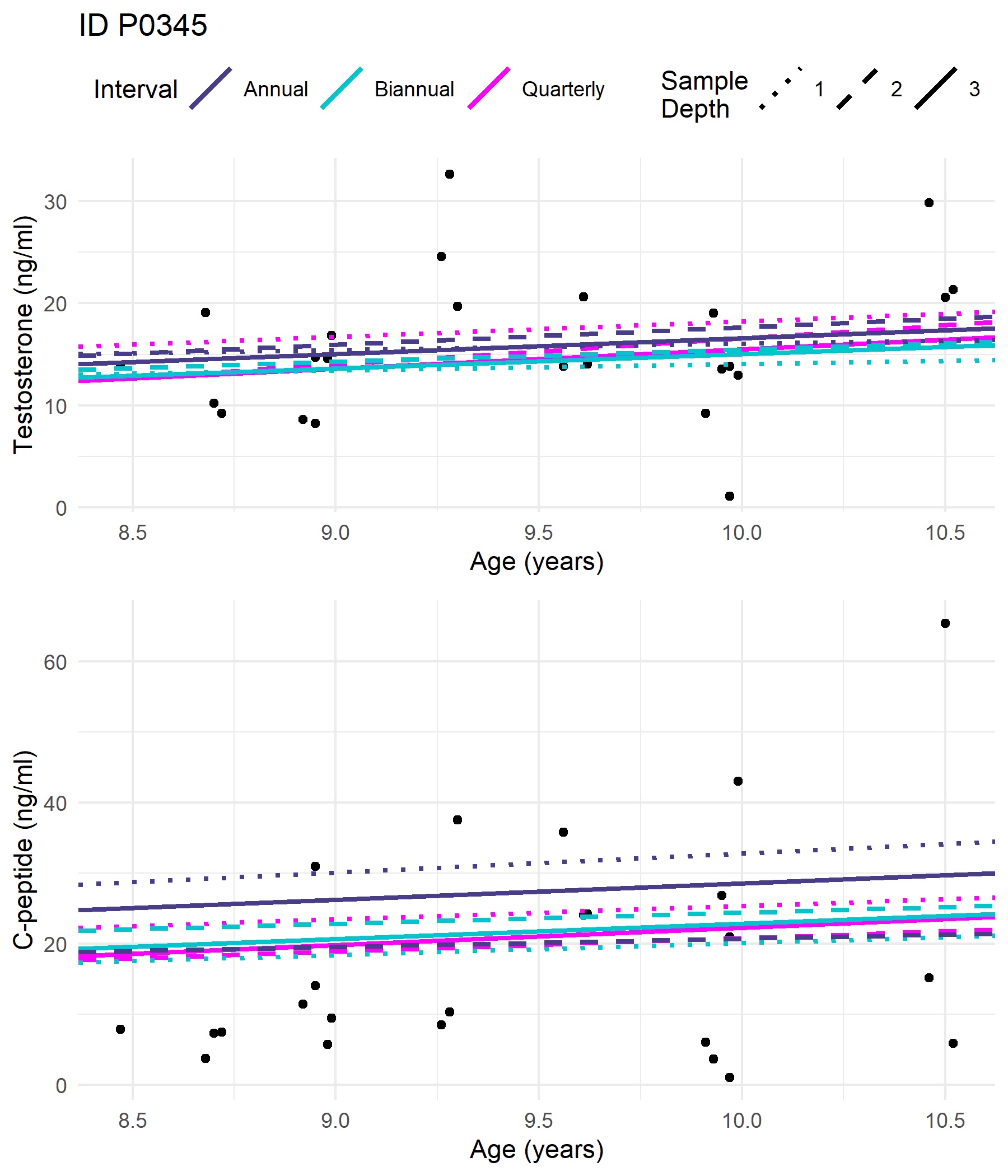


**Figure S4.** Observed biomarkers (3 samples per quarterly interval, n=27 testosterone and C-peptide) for one individual across ages 8.5-10.5 years with linear mixed model-estimated trends overlaid.


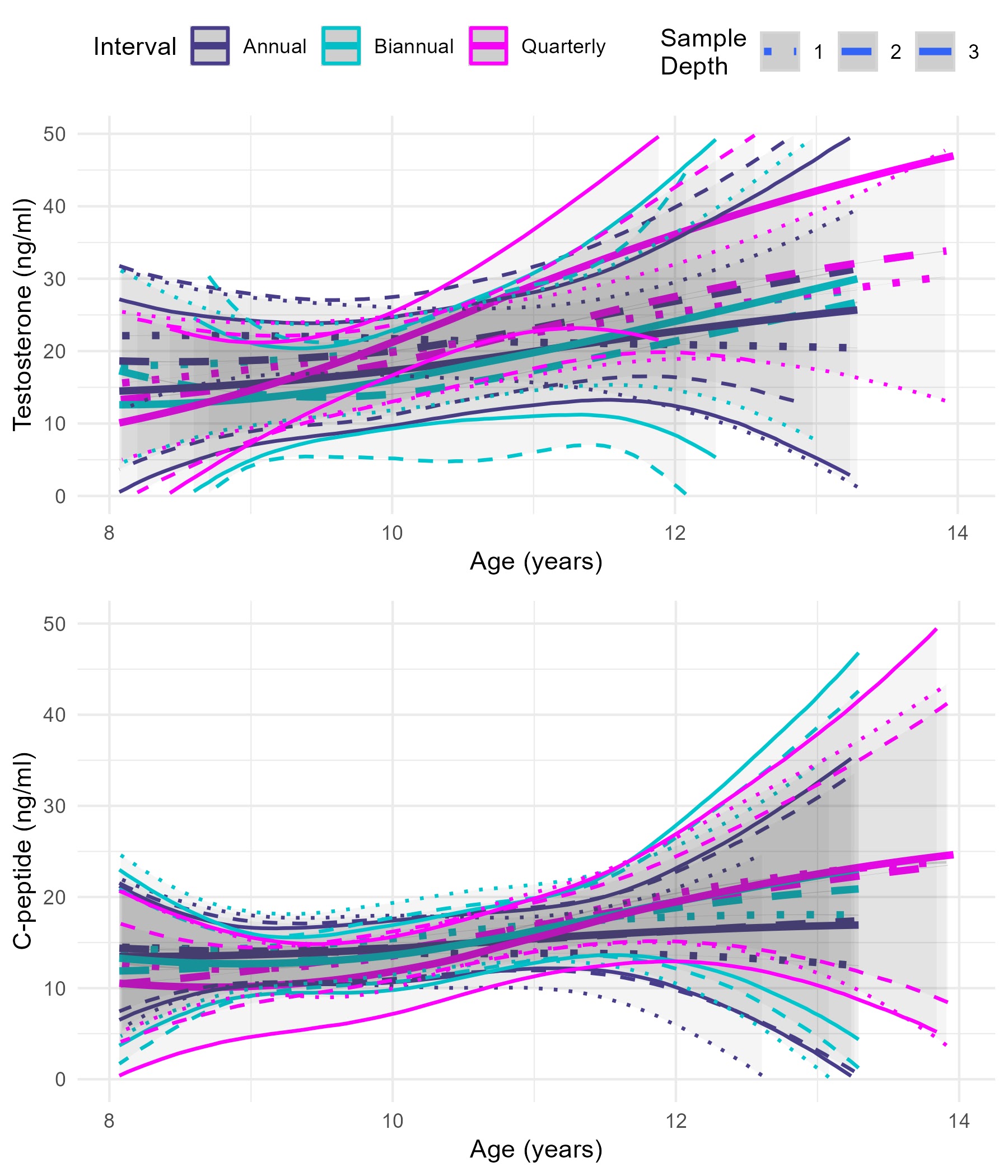


**Figure S5.** Population-level estimates in testosterone (top) and C-peptide (bottom) nonlinear trends across age. Mean lines and shaded 95% credible intervals overlay spline estimates from nine nonlinear generalized additive models with varying sampling frequencies (quarterly, biannual, and annual intervals with 1, 2, and 3 samples per interval).

**
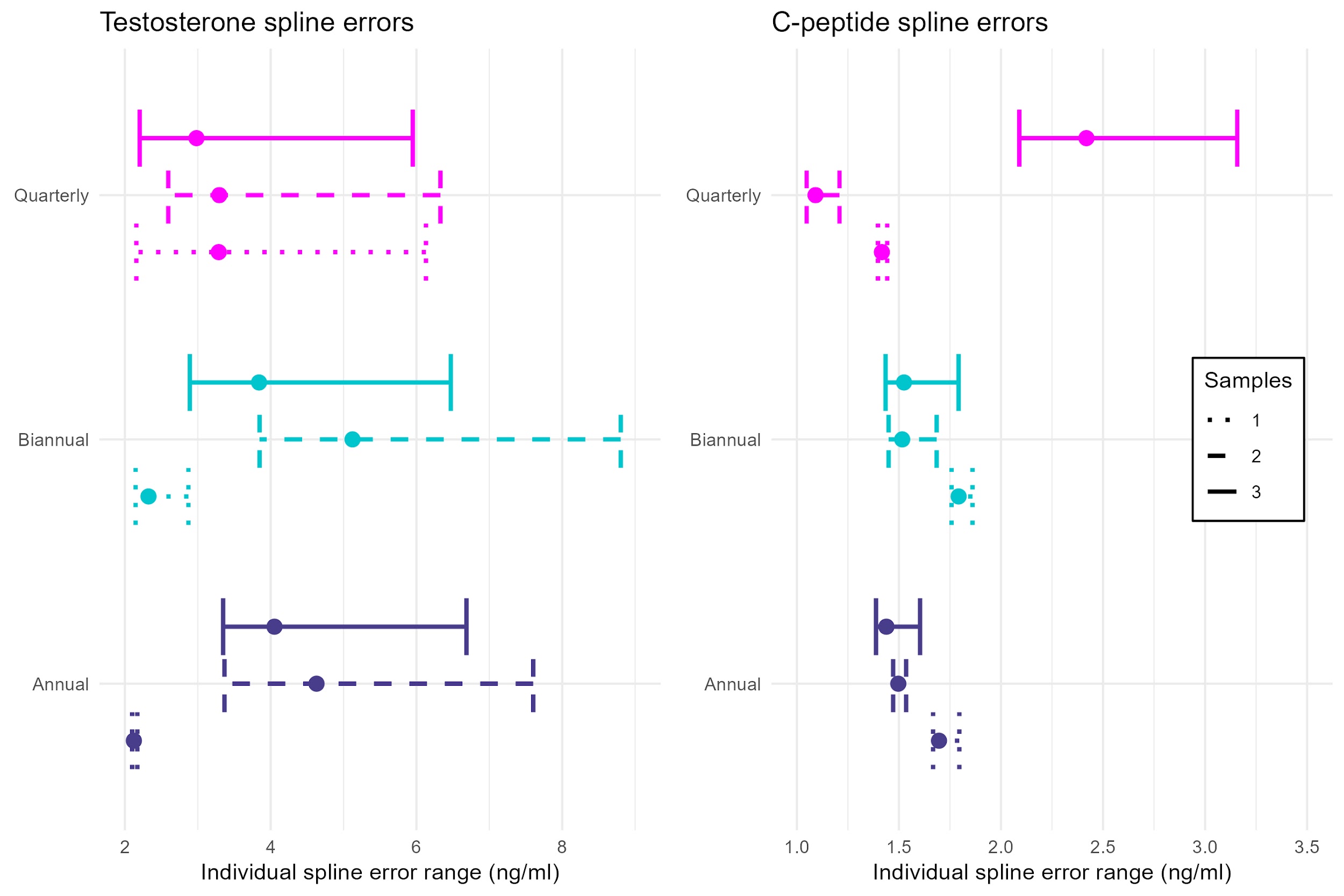
**

**Figure S6.** Means and full ranges of testosterone (left) and C-peptide (right) standard errors from individual spline estimates across age (n=35) in nine nonlinear generalized additive models with varying sampling frequencies (quarterly, biannual, and annual intervals with 1, 2, and 3 samples per interval).

**Table S1.** Literature summary table of pubertal testosterone and C-peptide/insulin studies. Overview of study scope, biomarkers collected, and sampling criteria.

| **Study Reference** | **Study Scope** | **Hormone (Specimen)** | **Study Type** | **Sampling Frequency & Depth** | **Sampling Strategy & Inclusion Criteria** |
| --- | --- | --- | --- | --- | --- |
| Amiel et. al 1991 | Examine IR in puberty | Insulin & C-Peptide (fasting serum) | CS | Continuous infusion ~90 mins, 6x/~2hrs on 1 sampling occasion | Females & males pre/post pubertal by TS & T/E cut-offs |
| Ankarberg & Norjavaara 1999 | Examine pubertal T change | T (serum) | LNG | 7x / 24hrs | Females aged 4.6-16.5, Prepubertal -late puberty |
| Apter 1980 | Monitor pubertal hormone & physical changes | T (serum) | LNG | 3x | Females ages 7-17 at baseline, followed up at 1 & 1.5 yr |
| Apter & Vihko 1977 | Clarify pubertal hormone changes | T (serum) | CS | 1x | Females aged 7-17 by TS, bone age, & gynecological age |
| Ball et al 2006 | Determine pubertal SI | Insulin (fasting serum) | LNG | 2x sampling occasions 18x/~2hrs | Children (>= TS 1) & ages 4-12 yr at baseline, follow up 2yr |
| Biro et al 2019 | Examine sex hormones & physical maturation | T (fasting morning serum) | LNG | 12x (1x every 6 months across 6 yr) | Females & males ages 6-7 at baseline, followed for 6 yr |
| Caprio et. al 1989 | Assess IR in puberty | Insulin & C-Peptide (fasting serum) | CS | 16x/~2hr | Females & males ages 8-17, TS “preadolescent” & “adolescent” |
| Chavarro et. al 2017 | Compare & test types of pubertal assessments | C-Peptide (fasting serum) | CS | 1x | Females & males ages 8-13, pubertal stage questionnaire, clinician TS. Hormone analysis |
| Cook et. al 1993 | Examine pubertal SI & other hormones | T & Insulin (fasting serum) | CS | 1x | Females & Males according to TS & T/E hormonal cut-offs |
| Cuartero et. al 2007 | Establish reference values | Insulin & C-Peptide (fasting serum) | CS | 1x | Females & males ages 1 month-18 yr old, TS |
| Goran & Gower 2001 | Assess pubertal SI | Insulin & C-Peptide (fasting serum) | LNG | 2x | Females & males baseline TS I, follow up at TS III-IV |
| Garcés et al 2010 | Examine sex hormones & other metabolic markers | T (fasting serum) | CS | 1x | Females & males ages 12-15 |
| Hannon et. al 2006 | Validate prior work on pubertal IR & anthropometrics | Insulin & C-Peptide (fasting serum) | LNG | 4x total (2x/1-3 weeks a part) | Females & males baseline TS & T/E cut-offs |
| Hoekstra et. al 2012 | Estimate heritability of T | T (Midday salivary) | LNG | 2x | Female & male twins age 12, TS |
| Jeffrey et al 2012 | Establish relation between pubertal IR & other factors | Insulin (fasting serum) | LNG | Annually 9x | Females & males ages 5-14, TS, & LH in detectable limits |
| Kelly et al 2011 | Examine pubertal changes in SI, AIR, & DI | Insulin (fasting serum) | LNG | 11x/~2.5hrs, annual 5x | Females & males ages 8-13 at baseline, TS, followed for 5 yr |
| Kelsey et al 2020 | Assess pubertal SI & insulin secretion | Insulin & T (fasting serum) | LNG | 3x | Females & males TS2-3 baseline until completion of puberty |
| Kim et al 2021 | Examine adiposity & sex steroids | Insulin & T (fasting serum) | LNG | 1x (insulin) 2x (T) | Females & males ages 10 & 17 |
| King et al 2020 | Assess pubertal hormonal coupling | T (morning salivary) | LNG | 2x | Females & males ages 9-13 & TS 1 at baseline, 2 yr follow up at TS 2 |
| Marceau et al 2014 | Assess pubertal hormonal coupling & social factors | T (serum) | LNG | 3x/hr every 6 months, 3x total | Females & males in two samples aged 9-14 or 11-16 |
| Marceau et al 2015 | Examine hormonal coupling | T (salivary) | LNG | 3x per day for 3 days | Females & males aged 11-16 |
| Matchock et al 2007 | Examine diurnal hormonal variation | T (salivary) | LNG | 5x/day (15x total) | Females (ages 8,10,12) & males (9,11,13), 3 waves 6 months apart |
| McCartney et al 2007 | Assess hyperandrogenemia & sex steroids | T & Insulin (fasting serum) | CS | 1x | Female’s age 7-17, TS |
| Mitamura et al 2000 | Investigate diurnal hormonal changes before puberty | T (serum) | LNG | Every 20 mins across 24hrs (96x) | Female’s age 5.1-11.4, pre-pubertal & pubertal, T/E/FSH cut points |
| Moran 1999 | Characterize IR in puberty | Insulin (fasting serum) | CS | 3x after infusion on 1 sampling occasion | Females & males ages 8-14, TS |
| Nokoff 2019 | Examine obesity & sex hormones | Insulin (fasting serum) | CS | 1x | Females & males ages 9+, TS2-3 at recruitment |
| Perng et al 2018 | Examine metabolic risk across adolescence | C-Peptide (fasting serum) | LNG | 2x | Females & males ages 8-14 at baseline, follow up 5 yr later, pubertal (TS>1) & pre-pubertal |
| Phan et. al 2021 | Examine stress & psychopathologies on hormonal coupling | T (morning saliva) | LNG | 3x/day across 3 days at time point 2 | Females & male twin pairs, ages 8 (at time 1) & age 13 (time point 2), TS self & maternal report at time 2 |
| Shirtcliff et al 2009 | Assess pubertal hormones & pubertal report | T (saliva) | CS | 8x/day | Females & males ages 9-14, PDS, physical & picture-based exam |
| Simmons et al 2015 | Examine childhood adversity & sex/steroid hormones | T (waking saliva) | LNG | 2x across 2 days | Females & males ages 12.41, 14.91, & 15.56, TS1-3 |
| Singh et al 2015 | Describe temporal changes in pubertal hormones | Insulin (fasting serum) | LNG | 3x across 12 months | Females & males ages 10-12, by TS |
| Travers et al 2002 | Assess risk factors for fat accumulation | Insulin (fasting serum) | LNG | 13x/~2.5hrs, 4x total | Females & males ages 9.7-14.5, baseline TSII-III, follow up yr 2 & 3 |
| Turan et al 2015 | Evaluate cortisol-T coupling & social evaluative threat | T (saliva) | CS | 4x across 1 day | Females & males ages 8-11, baseline & post-stress test collection |
| Vijayakumar et al 2019 | Examine pubertal affective reactivity & T | T (morning saliva) | LNG | 3x | Females & males, ages 9-11 baseline, PDS, 3 waves 3 yr apart |
| Watkins et al 2016 | Test urinary metabolites, C-peptide & IGF-1 | Insulin (fasting serum) | CS | 1x | Females & males ages 8-14, pubertal if TS>1 |
| Wierenga et al 2018 | Characterize changes in pubertal subcortical brain volumes in relation to hormones | Testosterone (morning saliva) | LNG | 3x | Females & males ages 8-29, PDS, 3 waves total with 2-year intervals |

**Legend**

LNG= Longitudinal design

CS= Cross-sectional design

T= Testosterone

SI= Insulin Sensitivity

DI= Insulin Disposition Index

AIR= Acute Insulin Response

PDS= Pubertal Development Scale

TS= Tanner Stage (I-V)

IR= Insulin Resistance

**Table S2.** Bayesian generalized additive model results from nine sampling frequencies of testosterone and C-peptide. Individual statistics summarize 35 splines for each individual’s nonlinear hormone trend across age. Population level spline statistics include 95% credible intervals (2.5% CI/97.5% CI) for the average nonlinear age trends across all individuals. Spline errors reflect the standard deviation of each parameter’s posterior distribution. Lambda is a smoothing hyperparameter estimated to fit each spline, with larger values indicating smoother, more linear trends, and sigma indicates the standard deviation of model residuals.
